# Impact of Rituximab on Immunoglobulin Concentrations and B Cell Numbers after Cyclophosphamide Treatment in Patients with ANCA-Associated Vasculitides

**DOI:** 10.1371/journal.pone.0037626

**Published:** 2012-05-21

**Authors:** Nils Venhoff, Nora M. Effelsberg, Ulrich Salzer, Klaus Warnatz, Hans Hartmut Peter, Dirk Lebrecht, Michael Schlesier, Reinhard E. Voll, Jens Thiel

**Affiliations:** 1 Department of Rheumatology and Clinical Immunology, University Hospital Freiburg, Freiburg, Germany; 2 Centre for Chronic Immunodeficiency (CCI), University Hospital Freiburg, Freiburg, Germany; Institut Jacques Monod, France

## Abstract

**Objective:**

To assess the impact of immunosuppressive therapy with cyclophosphamide (CYC) and rituximab (RTX) on serum immunoglobulin (Ig) concentrations and B lymphocyte counts in patients with ANCA-associated vasculitides (AAVs).

**Methods:**

Retrospective analysis of Ig concentrations and peripheral B cell counts in 55 AAV patients.

**Results:**

CYC treatment resulted in a decrease in Ig levels (median; interquartile range IQR) from IgG 12.8 g/L (8.15-15.45) to 9.17 g/L (8.04-9.90) (p = 0.002), IgM 1.05 g/L (0.70-1.41) to 0.83 g/L (0.60-1.17) (p = 0.046) and IgA 2.58 g/L (1.71-3.48) to 1.58 g/L (1-31-2.39) (p = 0.056) at a median follow-up time of 4 months. IgG remained significantly below the initial value at 14.5 months and 30 months analyses. Subsequent RTX treatment in patients that had previously received CYC resulted in a further decline in Ig levels from pre RTX IgG 9.84 g/L (8.71-11.60) to 7.11 g/L (5.75-8.77; p = 0.007), from pre RTX IgM 0.84 g/L (0.63-1.18) to 0.35 g/L (0.23-0.48; p<0.001) and from pre RTX IgA 2.03 g/L (1.37-2.50) to IgA 1.62 g/L (IQR 0.84-2.43; p = 0.365) 14 months after RTX. Treatment with RTX induced a complete depletion of B cells in all patients. After a median observation time of 20 months median B lymphocyte counts remained severely suppressed (4 B-cells/µl, 1.25-9.5, p<0.001). Seven patients (21%) that had been treated with CYC followed by RTX were started on Ig replacement because of severe bronchopulmonary infections and serum IgG concentrations below 5 g/L.

**Conclusions:**

In patients with AAVs, treatment with CYC leads to a decline in immunoglobulin concentrations. A subsequent RTX therapy aggravates the decline in serum immunoglobulin concentrations and results in a profoundly delayed B cell repopulation. Surveying patients with AAVs post CYC and RTX treatment for serum immunoglobulin concentrations and persisting hypogammaglobulinemia is warranted.

## Introduction

The group of ANCA-associated vasculitides (AAVs) comprises granulomatosis with polyangiitis (GPA, Wegener’s granulomatosis), microscopic polyangiitis (MPA) and Churg-Strauss syndrome (CSS). Since 1971 cyclophosphamide (CYC) has been the standard treatment for severe, life-threatening AAVs [1]. These diseases are histologically characterized by a necrotizing inflammation of small vessel walls mediated by ANCAs and cytokine primed neutrophils [2]. Cytokine-primed neutrophils, antineutrophil cytoplasmic antibodies (ANCA) and B lymphocytes play a significant role in the pathogenesis of AAVs [3]. The pathogenic role of B lymphocytes in AAVs is emphasized by the observation of increased concentrations of BAFF in the serum of patients with GPA [4]. Furthermore, B lymphocyte targeted therapy with rituximab (RTX) has been shown to be effective in the induction therapy of AAVs, as well as in the treatment of relapsing AAV disease activity [5]–[7]. The standard induction therapy regimen with CYC bears the risk of infections, infertility and malignancy. Only very limited data are available evaluating the effect of a combined therapy with CYC and RTX on peripheral B lymphocyte counts and immunoglobulin concentrations over a prolonged observation period. Such data are of considerable interest since both therapies can potentially induce hypogammaglobulinemia leading to an increased risk of infections [8]. Microbial factors in turn may induce vasculitic flares, worsening the overall disease outcome [9], [10]. Here, we report on changes in serum Ig concentrations, peripheral B cell numbers and infectious complications in AAV treated with CYC or CYC followed by RTX.

## Methods

### Inclusion Criteria

Patients recruited for this retrospective analysis regularly attended the Department of Rheumatology, University Hospital Freiburg. Inclusion in the analysis required a diagnosis of ANCA-associated vasculitis (GPA, MPA, or CSS) that had been treated with CYC or CYC and RTX. After ethics committee approval (ethic committee of the Albert-Ludwigs-University, Freiburg, EC Freiburg 191/11, 46/04) written informed consent was obtained and the patients’ clinical charts were retrospectively analysed. 72 patients (32 females, 40 males) were classified as AAV (GPA, n = 58; MPA, n = 5; CSS, n = 9) according to the American College of Rheumatology and the Chapel Hill Consensus Criteria and had been treated with CYC or CYC and RTX [11]–[13]. Patients treated with RTX and fewer than 6 months follow-up were excluded from the analysis (n = 2), as were patients with incomplete data set precluding immunoglobulin (Ig) or peripheral blood B lymphocyte analysis (n = 14). One patient had to be excluded because of nephrotic syndrome (n = 1) at time of Ig analysis potentially affecting serum immunoglobulin concentrations. In this patient no data on B cells after RTX were available. Fifty-five patients (24 females, 31 males) were included in the study. The majority had GPA (n = 44), seven had CSS, and four MPA. 91% of the patients were ANCA positive. Median age was 57 years (age range 27–79 years). For more details see Table 1. Substitution of immunoglobulins or plasmapheresis during follow-up led to exclusion of the patient from follow-up immunoglobulin analyses.

**Table 1:** Patients’ characteristics of the AAV cohort.

	All patients	CYC#	CYC+RTX	p value
**Patients’ characteristics**				
Number, n (female/male)	55 (24/31)	36 (16/20)	33 (14/19)	0.872
Age, median years (IQR)	57 (48–69.75)	58.5 (49.5–68.5)	56 (47–68.25)	0.815
Disease duration, median months (IQR)	78 (44.75–120.75)	89 (55.5–117.5)	72 (40.25–168.75)	0.564
**Diagnosis, n (%)**				
GPA	44 (80.0)	30 (28)	28 (84.8)	0.872
MPA	4 (7.3)	2 (5.6)	2 (6.1)	0.792
CSS	7 (12.7)	4 (11.1)	3 (9.1)	0.925
**ANCA status, n (%)**				
ANCA positive	50 (90.9)	33 (91.7)	31 (93.9)	0.728
PR3-ANCA positive	38 (69.1)	25 (69.4)	26 (78.8)	0.385
MPO-ANCA positive	6 (10.9)	4 (11.1)	3 (9.1)	0.792
**Disease stage, n (%)**				
Localised	4 (7.3)	4 (11.1)	1 (3.0)	0.204
Systemic	51 (92.7)	32 (88.9)	32 (97.0)	0.204
**Organ involvement, n (%)**				
Renal	25 (45.5)	13 (36.1)	19 (57.6)	0.077
Pulmonary	42 (76.4)	26 (72.2)	26 (78.8)	0.536
Ear, nose, throat (ENT)	42 (76.4)	27 (75.0)	28 (84.8)	0.317
Skin	14 (25.5)	8 (22.2)	8 (24.2)	0.850
Central nervous system	7 (12.7)	5 (13.9)	5 (15.2)	0.890
Peripheral nervous system	22 (40.0)	11 (30.6)	15 (45.5)	0.208

Characteristics of patients with ANCA-associated vasculitis analysed for effects of cyclophosphamide (CYC) and rituximab (RTX).

GPA, granulomatosis with polyangiitis (Wegener’s granulomatosis); MPA, microscopic polyangiitis; CSS, Churg-Strauss syndrome; ANCA, antineutrophil cytoplasmic antibody; PR3, proteinase3; MPO, myeloperoxidase; IQR, interquartile range; CYC, cyclophosphamide; RTX, rituximab.

If not indicated otherwise, median and interquartile range are reported;

# 14 patients of CYC analysis group were later treated with RTX and subsequently also enrolled into the RTX analysis group.

### AAV Patients Treated with Cyclophosphamide

Thirty-six patients were analyzed for CYC effects, 30 patients with a diagnosis of GPA, four with CSS, and two with MPA. Data from 32 of these patients were available for serum immunoglobulin analyses and data from 22 patients were available for B cell analyses. All but three patients were ANCA positive. Detailed patients’ characteristics are presented in table 1. The cumulative CYC dose was 7.88 gram (g). Table 2 summarizes the different cumulative immunosuppressive therapy regimens before and after the application of CYC. Intravenous immunoglobulins and plasmapheresis were used in one patient each as immunomodulatory therapies. One patient did not receive immunosuppressive maintenance therapy after cessation of CYC treatment. Fourteen patients of the CYC analysis group were at a later time point treated with RTX and therefore also enrolled into the RTX analysis group.

**Table 2:** Treatment characteristics of the AAV cohort.

	All patients (n = 55)	CYC (n = 36)#	CYC + RTX (n = 33)	p value
**Prednisone (mg/day) within the four week interval prior to treatment with CYC or RTX**				
CYC, median (IQR)	0.0 (0.0–15.0)	0.0 (0.0–18.75)	5.0 (0.0–11.25)	0.755
CYC, mean (SD)	8.8 (±12.8)	8.0 (±11.3)	8.4 (±12.8)	
RTX, median (IQR)	n.a.	n.a.	7.5 (5.0–12.5)	
**Prednisone dose (mg/day) 6, 12, and 24 months after CYC or RTX treatment**				
6 months, median (IQR)	n.a.	7.5 (7.5–13.75)	10.0 (7.5–15.0)	0.527
12 months, median (IQR)	n.a.	7.5 (6.875–10.0)	7.5 (5.0–7.5)	0.552
24 months, median (IQR)	n.a.	5.0 (5.0–8.75)	5.0 (5.0–7.5)	0.587
**CYC and RTX (cumulative dose in gram)**				
CYC dose, median (IQR)	n.a.	7.88 (5.525–20.0)	14.45 (9.375–38.75)	**0.012**
RTX dose, median (IQR)	n.a.	n.a.	2.0 (1.0–5.0)	n.a.
One/two/three courses, n			15/12/6	
**Induction therapy, n (%)**				
CYC	43 (78.2)	26 (72.2)	27 (81.8)	0.353
MTX	6 (10.9)	5 (13.8)	4 (12.1)	0.837
AZA	3 (5.5)	3 (8.3)	0 (0)	0.096
MMF	2 (3.6)	1 (2.8)	2 (6.1)	0.518
CSA	1 (1.8)	1 (2.8)	0 (0)	0.353
**Cumulative treatment before start of CYC, n (%)**				
Prednisone	27 (49.1)	16 (44.4)	18 (54.5)	0.409
MTX	8 (14.5)	7 (19.4)	5 (15.2)	0.647
AZA	4 (7.3)	3 (8.3)	1 (3.0)	0.358
MMF	2 3.6)	1 (2.8)	2 (6.1)	0.518
LEF	3 (5.5)	3 (8.3)	2 (6.1)	0.728
CSA	2 (3.6)	1 (2.8)	1 (3.0)	0.967
TNFα antagonist	2 (3.6)	2 (5.6)	1 (3.0)	0.622
no immunosuppressive agent*	43 (78.2)	26 (72.2)	27 (81.8)	0.353
**Cumulative treatment after start of CYC and prior to RTX treatment, n (%)**				
Prednisone	55 (100)	36 (100)	33 (100)	1.000
MTX	34 (61.8)	21 (58.3)	20 (60.6)	0.854
AZA	34 (61.8)	18 (50.0)	22 (66.7)	0.166
MMF	19 (34.5)	7 (19.4)	15 (45.5)	**0.022**
LEF	16 (29.1)	10 (27.8)	11 (33.3)	0.624
CSA	3 (5.5)	1 (2.8)	2 (6.1)	0.518
TNFα antagonist	7 (12.7)	4 (11.1)	5 (15.2)	0.628
**Cumulative treatment after RTX treatment, n (%)**				
MTX	n.a.	n.a.	9 (27.3)	
AZA	n.a.	n.a.	10 (30.3)	
MMF	n.a.	n.a.	9 (27.3)	
LEF	n.a.	n.a.	6 (18.2)	
Previous immunotherapy, mediannumber (IQR)‡	n.a.	3.0 (2–4)	3.0 (1–4)	0.431

Treatment characteristics of all AAV patients, patients treated with CYC and patients treated with RTX after previous CYC therapy.

CYC, cyclophosphamide; RTX, rituximab; MTX, methotrexate; AZA, azathioprine; MMF, mycophenolate mofetil; LEF, leflunomide; CSA, cyclosporine A; IQR, interquartile range.

* other than prednisone,

‡ before CYC or RTX treatment,

# 14 patients of the CYC group were later treated with RTX and subsequently also enrolled in the RTX analysis group.

### AAV Patients Treated with Rituximab after Previous CYC Therapy

Thirty-three patients of the study group were treated with RTX subsequently to CYC. Patients were treated with RTX as off-label therapy for refractory disease or had relapsed after standard immunosuppressive therapy including CYC. Detailed characteristics of these patients and the immunsuppressive treatment are listed in table 1 and table 2. Cumulative CYC dose was 14.45 g. Data from 18 patients were analysed for changes in serum Ig concentrations after RTX treatment. In these patients serum Ig concentrations immediately prior to RTX application and during follow-up were available. Twenty-four RTX treated patients were included in the analysis of peripheral B cell numbers.

### Clinical and Laboratory Assessments

Data analyses of these patients comprised laboratory values, evaluation of disease extent and organ involvement. All disease stages were defined according to EULAR/EUVAS [14]. Ig serum concentrations (normal range: IgG 7-16 g/L, IgM 0.4-2.3 g/L, IgA 0.7-4 g/L) were determined by nephelometry (Behring Nephelometer). The ANCA staining pattern (cytoplasmatic or perinuclear) was assessed by indirect immunofluorescence. ANCA’s specificity for proteinase 3 (PR3) (Organtec) or myeloperoxidase (MPO) (Euroimmun) was measured by enzyme-linked immunosorbent assay (ELISA) and interpreted according to manufacturers’ reference ranges with the upper limit of the normal of <10 U/ml for PR3 and <20 U/ml for MPO. Peripheral B lymphocytes were analyzed by 4-colour flow cytometry (FACSCalibur, BD-Biosciences) [15]. B cell depletion was defined as counts that were ≤2 B cells/µl.

### Vaccination Response to Pneumococcal Polysaccharides

Four RTX treated patients were vaccinated against pneumococcal polysaccharides (Pneumovax®). Specific IgG antibodies were assayed by using a serotype-specific (serotypes 4, 5, 6B, 7F, 9V, 14, 18C, 19F, and 23F) ELISA. The assays were performed according to World Health Organization recommendations with reference serum 89-SF as standard [16].

### Statistical Analysis

Results were compared by Wilcoxon-analysis using SigmaStat software version 11.0 (Systat Software, Inc. SigmaStat for Windows). All statistical tests were two-tailed and a p-value <0.05 was considered statistically significant. When not otherwise indicated median and interquartile range (median; IQR) are reported.

## Results

### Patients’ Characteristics and Treatment

There was no difference in age, sex, ANCA status, organ involvement, disease extent and duration between the patient groups (Table 1). Daily prednisone doses at the time of serum immunoglobulin measurement and median number of immunosuppressive agents were comparable. The cumulative CYC dose was significantly higher in patients treated with RTX (p = 0.012) compared to patients treated with CYC only. Patients were treated with RTX when AAV disease activity was resistant to CYC treatment. We analysed serum immunoglobulin concentrations immediately prior to CYC and RTX therapy. Application of an additional course of RTX, substitution of immunoglobulins or plasmapheresis during follow-up led to exclusion from follow-up B lymphocyte or immunoglobulin analyses. Ig concentrations (g/L) prior to application of CYC as well as prior to subsequent application of RTX were within normal range (Ig concentrations prior to CYC: IgG 12.8 g/L, IQR 8.15-15.45; IgM 1.05 g/L, IQR 0.70-1.41; IgA 2.58 g/L, IQR 1.71-3.48; Ig concentrations prior to RTX: IgG g/L 9.84, IQR 8.71-11.60; IgM g/L 0.84, IQR 0.63-1.18; IgA g/L 2.03; IQR 1.37-2.50). There was no association between low serum immunoglobulin concentrations and renal dysfunction (as defined by creatinine >1.17 mg/dl (p = 0.952) or a decrease in the glomerular filtration rate calculated by Cockroft-Gault). Peripheral B lymphocytes were measured one month before and four, respectively 20 months after RTX and 20 months after CYC treatment.

### Effect of Cyclophosphamide on Serum Immunoglobulin Concentrations and Peripheral B Cell Numbers

**Figure 1:**
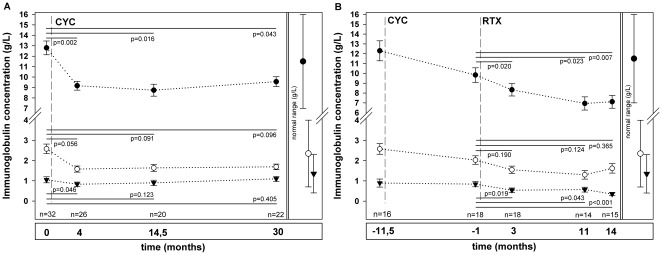
Effect of CYC treatment and CYC treatment followed by RTX on serum Ig concentrations in AAV patients. Vertical dashed lines indicate the time of treatment with CYC (**A**) and CYC followed by RTX (**B**). Black circles stand for IgG concentrations, open circles for IgA concentrations, and black triangles for IgM concentrations. Vertical bars on the right represent the normal ranges of IgG, IgA and IgM serum concentrations. Median ± SE are reported.

The effect of CYC on serum immunoglobulin concentrations was assessed in 32 patients. Serum immunoglobulin concentrations were analyzed at median 4 months (n = 26 patients), 14.5 months (n = 20 patients) and 30 months (n = 22 patients). Four months after CYC treatment IgG and IgM concentrations had decreased significantly (IgG 9.17 g/L, IQR 8.04-9.90, p = 0.002; IgM g/L 0.83, IQR 0.60-1.17, p = 0.046; IgA 1.58 g/L, IQR 1-31-2.39, p = 0.056) and IgG remained significantly below the initial value at 14.5 months analysis (IgG 8.74 g/L, IQR 7.43-10.54, p = 0.016; IgM 0.90 g/L, IQR 0.43-1.175, p = 0.123; IgA 1.65 g/L, IQR 1.10-2.165, p = 0.091) and 30 months analysis (IgG 9.56 g/L, IQR 8,43-11.0, p = 0.043; IgM 1.105 g/L, IQR 0.77-1.38, p = 0.405; IgA 1.69 g/L, IQR 1.12-2.25, p = 0.096) (Figure 1a). Median number of peripheral B lymphocytes 19.5 months after CYC treatment was 62/µl (IQR 36-102/µl).

### Effect of Rituximab on Serum Immunoglobulin Concentrations after Previous CYC Treatment

The treatment with RTX after previous CYC exposure resulted in a further significant decline in Ig concentrations at 3 month’s analysis from 9.84 g/L (IQR 8.71-11.60) to 8.34 g/L (IQR 6.32-9.80), and in IgM from 0.84 g/L (IQR 0.63-1.18) to 0.54 g/L (IQR 0.31-0.80) (p = 0.02 and p = 0.019). The decline in IgA from 2.03 g/L (IQR 1.37-2.50) to 1.55 g/L (IQR 1.07-2.29) did not reach statistical significance (p = 0.190) (Figure 1 b). The analyses at 11 months showed comparable results with a significant decrease in IgG and IgM (p = 0.023 and p = 0.043), while the decrease in IgA was not significant (p = 0.124). After 14 months IgG had declined to 7.11 g/L (5.75-8.77; p = 0.007), IgM to 0.35 g/L (0.23-0.48; p<0.001) and IgA to 1.62 g/L (IQR 0.84-2.43; p = 0.365). In four patients the observation period after RTX treatment was more than 36 months. In one patient IgG concentrations returned to 12.8 g/L and peripheral B cell numbers to 154/µl after an initially complete B cell depletion. Two patients had persistently reduced IgG concentrations (5.57 g/L and 6.71 g/L) and in one patient Ig concentrations stabilized at 8.54 g/L.

Patients with subsequent RTX treatment were previously treated with a significantly higher cumulative CYC dose. Therefore, we tested whether higher CYC doses accounted for the more profound decrease in serum immunoglobulin levels after RTX treatment. We compared patients treated with CYC sum dose >15 g (median CYC 51 g, IQR 29.38-130.13) to patients with CYC sum dose <15 g (median CYC 8.25 g, IQR 6.63-12.38). The difference in the decline in serum Ig concentrations was statistically not significant between patients treated with low CYC doses (IgG 5.54 g/L; IgM 0.29 g/L; IgA 1.49 g/L) compared to those treated with high CYC doses (IgG 8.19 g/L, IgM 0.49 g/L; IgA 1.20 g/L; p = n.s. for all). After CYC treatment MMF was significantly more often used in patients that were subsequently treated with RTX (Table 2) and MMF was also more often used as maintenance therapy after RTX treatment (p = 0.022). To rule out that immunosuppressive therapy with MMF accounts for low serum immunoglobulin levels after RTX treatment, we excluded patients treated with MMF after RTX from analysis of serum immunoglobulin concentrations. Even when these patients were excluded a significant decrease in serum immunoglobulin concentrations after RTX treatment was observed (data not shown).

### Impaired Specific Antibody Responses and Infectious Complications in AAV Patients

**Figure 2:**
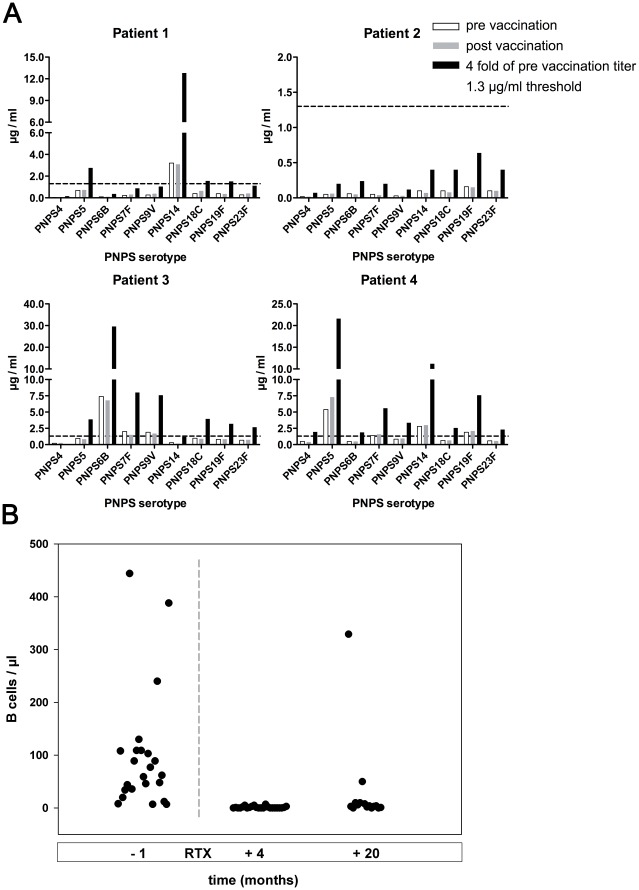
Influence of RTX on peripheral B cell numbers and specific antibody responses. (**A**) Pneumococcal polysaccharide (PnPs) response after vaccination. PnPs responses to nine individual serotypes (4, 5, 6B, 7F, 9V, 14, 18C, 19F, 23F) before (open bars) and 4 weeks after (grey bars) vaccination with 23-valent Pneumovax® vaccine in 4 patients. Black bars indicate calculated 4-fold increase of the pre-vaccination titer, the dashed line indicates the 1.3 µg/ml threshold according to interpretation guidelines of PnPs responses issued by the AAAAI [17]. (**B**) B cell depletion at median 4 and 20 months after treatment with RTX.

IgG concentrations in 18 of the CYC/RTX treated patients (54%) and six of the patients without RTX treatment (21%) dropped to serum levels below 7.0 g/L after therapy with RTX, respectively CYC. In the RTX analysis group seven patients (21%) had serum IgG levels even below 5.0 g/L (range: IgG 2.82–4.59 g/L) and required regular Ig substitution therapy because of severe bronchopulmonary infections independent of AAV activity. Three patients suffered from recurrent episodes of bacterial sinusitis and bronchitis. In three patients pneumonia with infiltrates on chest x-ray, elevated C-reactive protein and elevated procalcitonine was diagnosed, and responded rapidly to antibiotic treatment, while immunosuppressive therapy regimen was not changed. In one patient a mesenterial abscess formation with extended-spectrum B lactamase strains of Klebsiella pneumoniae and Escherichia coli occurred that prompted combined surgical and antibiotic treatment. This patient had previously been treated with high doses of prednisone. Pneumocystis pneumonia did not develop in any of the patients. Only one patient in the CYC analysis group suffered from recurrent respiratory tract infections not related to active vasculitis and was treated with immunoglobulin replacement therapy. The rationale for starting immunglobulin replacement therapy were persistently reduced serum Ig concentrations, recurrent infections and – if tested – an inadequate antibody response to vaccinations. In four RTX treated patients we assessed the specific antibody response after Pneumovax® vaccination. IgG antibody titers against nine individual serotypes were measured using an ELISA (Figure 2 a) based on the World Health Organization recommendation. Although 3 patients had significant titers for some pneumococcal polysaccharide (PnPS) serotypes prior to vaccination, there was almost no increase for any serotype in any patient after vaccination. In particular, none of the patients showed a greater than 4-fold increase for 3 serotypes. A seroconversion from titers below 1.3 µg/ml was absent for most pneumococcal polysaccharides. This indicates an impaired response according to published criteria [17]. On follow-up of the seven patients that had been started on immunoglobulin replacement therapy, only one of these patients returned to normal serum Ig concentrations.

### B Cell Reconstitution after Therapy with CYC and RTX

Peripheral B lymphocyte counts measured immediately before RTX were available in 23 patients, at four months analysis after RTX treatment in 24 and at 20 months analysis after RTX treatment in 15 patients. Application of additional courses of RTX led to exclusion from follow-up B lymphocyte analysis. Median B cell number prior to RTX therapy was 62/µl (IQR 34.5-108.75). Treatment with RTX resulted in a complete depletion of B cells (≤2/µl) in all patients within four months (median 0/µl, IQR 0-2, p<0.001). After a median observation time of 20 months (IQR 3.0-59.0) median B lymphocyte counts remained severely suppressed (median 4/µl, IQR 1.25-9.5, p<0.001) in 15 patients that were available for analysis (Figure 2 b). In five patients the median observation time was 30 months (26–51 months) and the median peripheral B cell number 8/µl. One of these patients had a complete reconstitution (154 B cells/µl), while three patients had B cell counts below 10/µl. CYC sum dose and frequency of MMF used as maintenance therapy after RTX had no significant influence on peripheral B lymphocyte numbers after the median observation time of 20 months (data not shown).

## Discussion

Recently, rituximab has been reported to be an efficacious and well tolerated treatment for AAVs [6], [18]–[20]. As CYC can deplete B lymphocytes, the long-term impact of RTX on serum immunoglobulins and the peripheral B lymphocyte compartment is of major interest in patients previously treated with CYC. In our patients treatment with CYC resulted in a decrease of all immunoglobulin isotypes, but median Ig concentrations remained within the normal range. We observed that particularly IgG and IgM isotypes of patients previously treated with CYC and subsequently receiving RTX further decreased significantly, whereas the drop in IgA serum concentration did not reach significance. In 54% of the CYC/RTX treated patients IgG declined to concentrations below 7.0 g/L and in 21% to concentrations below 5.0 g/L. In all patients with IgG concentrations below 5.0 g/L Ig replacement therapy had to be started because of clinically relevant infections. Furthermore, in four out of these patients testing for specific antibodies to pneumococcal polysaccharides revealed a defect in the antibody response. Our data indicate that in patients with AAVs that had previously been treated with CYC, RTX can lead to a further significant decline in serum immunoglobulin concentrations. The reasons for this finding are not clear. An additive effect of CYC and RTX as well as a synergy between RTX and maintenance immunosuppression might be causative. On the other hand, in our study maintenance immunosuppressive therapy was also used in patients that had only received CYC but no RTX. RTX induced hypogammaglobulinemia is rare in e.g. patients with rheumatoid arthritis (RA), and mainly affects the IgM isotype [20], [21]. This may indicate that there are indeed differences in Ig production and response to RTX in AAVs compared to other autoimmune diseases. In AAVs Ig production might be more dependent on a continuous differentiation of B cells into short-lived plasma cells, a process that would efficiently be targeted by RTX. Further studies with patients treated with RTX only have to address this issue. While not systematically assessed, some studies described a decrease in serum immunoglobulin concentrations after RTX therapy of AAV patients [22]–[25]. A recent survey of RTX therapy in 65 AAV patients comparable to our patients with regard to disease duration, previous immunosuppressive treatment and cumulative CYC doses reported a significant decrease in IgM concentrations [26]. Different from our results the median follow-up IgG concentration did not change significantly from baseline. However, no information is given on the exact time and the number of patients included into the IgG analysis. The reported IgG range of 1.5-28.9 g/L at baseline and 3.7-17.4 g/L at follow-up indicates profoundly decreased IgG concentrations in at least some patients. Interestingly, previous publications described a severe hypogammaglobulinemia in patients with lymphoma treated with CYC and RTX containing chemotherapies [27], [28]. Therefore our study is of considerable importance, as it is the first to report a statistically significant and clinically relevant decline in serum Ig concentrations in a substantial number of chronic AAV patients treated with CYC and RTX. The decrease in Ig concentrations is reflected by a prolonged depletion of B cells after CYC and RTX treatment. In patients with RA and Sjögren’s syndrome treated with RTX peripheral B cell reconstitution usually starts within six to nine months after therapy [29], [30]. Another retrospective analysis reported a repopulation of the peripheral B cell compartment by 11 months in 28 out of 45 patients [26]. Unfortunately, there is no information on absolute B cell numbers and the criteria for B cell depletion was set to <0.02×10^9^ cells/L, while ≥0.02×10^9^ B cells/L was the criteria for B cell repopulation. That rather indicates the period of time until B cells were again detectable but does not give sufficient information on reconstitution to normal of the peripheral B cell compartment. Our retrospective study has some limitations. Not all patients that were included into the study were available for Ig and B cell analyses at the analysed time points. The use of various different maintenance therapies after CYC and RTX – even while not statistically different between the groups – may still be a confounding factor. Furthermore, our study cannot provide a definite answer to the question whether there is a synergistic mode of action of RTX and CYC or maintenance immunosuppressive therapies. Larger cohorts of AAV patients with extended observation periods are needed to confirm our results and to assess long-term effects of CYC/RTX treatment on peripheral B cells and Ig serum concentrations. Furthermore, prospective, randomized trials have to answer at what time point AAV patients should be retreated with RTX, whether a critical cumulative CYC dose exists that should caution against subsequent RTX therapy, and whether maintenance immunosuppressive therapy is needed after RTX treatment.

In summary, we report on a long-lasting decrease of serum Ig and a retarded peripheral B cell reconstitution in AAV patients treated with RTX after previous CYC therapy. Therefore, in AAVs treated with CYC and RTX serum Ig concentrations and B cell counts need to be closely monitored. In case of increased infections in patients with secondary hypogammaglobulinemia immunoglobulin replacement therapy should be considered. RTX monotherapy needs to be evaluated for its effect on the humoral immunity of patients with AAVs and might be a treatment alternative to a dual therapy with CYC and RTX.
